# Mechanochemical Synthesis of BaTiO_3_ Powders and Evaluation of Their Acrylic Dispersions

**DOI:** 10.3390/ma13153275

**Published:** 2020-07-23

**Authors:** Sonia Kudłacik-Kramarczyk, Anna Drabczyk, Magdalena Głąb, Piotr Dulian, Rafał Bogucki, Krzysztof Miernik, Agnieszka Sobczak-Kupiec, Bożena Tyliszczak

**Affiliations:** 1 Institute of Materials Science, Faculty of Materials Engineering and Physics, Cracow University of Technology, 37 Jana Pawła II Av., 31–864 Krakow, Poland; magdalenaglab@op.pl (M.G.); rbogucki@mech.pk.edu.pl (R.B.); kmiernik@pk.edu.pl (K.M.); agnieszka.sobczak-kupiec@pk.edu.pl (A.S.-K.); 2 Institute of Inorganic Chemistry and Technology, Faculty of Chemical Engineering and Technology, Cracow University of Technology, 24 Warszawska St., 31–155 Krakow, Poland; piotr.dulian@pk.edu.pl

**Keywords:** barium titanate, perovskite powders, mechanochemical synthesis, acrylic poly(ethylene glycol) dispersions, microwave irradiation, sedimentation process

## Abstract

Barium titanate is a ferroelectric perovskite with unique electric properties; therefore, it is widely applied in the fabrication of inorganic coatings or thin films, capacitors, or in the production of devices for energy storage and conversion. This paper describes the mechanochemical synthesis of BaTiO_3_ from BaO and TiO_2_ using a ball mill. XRD analysis allowed concluding that barium titanate was formed after 90 min of mechanochemical grinding. It was also proved by spectroscopic analysis and the band corresponding to Ti–O vibrations on obtained Fourier Transform Infrared (FT-IR) spectra. The specific surface area of obtained powder was 25.275 m^2^/g. Next, formed perovskite was dispersed in an acrylic poly(ethylene glycol) (superabsorbent polymer suspension, SAP) suspension prepared using microwave radiation. Final suspensions differed in the concentration of SAP applied. It was proven that the increase of SAP concentration does not affect the acidity of the suspension, but it does increase its dynamic viscosity. A sample with 83 wt.% of SAP reached a value of approximately 140 mPa∙s. Dispersions with higher values of SAP mass fraction exhibited lower sedimentation rates. Molecules such as SAP may adsorb to the surface of particles and thus prevent their agglomeration and make them well monodispersed. Based on the performed experiments, it can be concluded that acrylic poly(ethylene glycol) suspension is a suitable fluid that may stabilize barium titanate dispersions.

## 1. Introduction

Barium titanate is an inorganic compound that due to its interesting properties has attracted particular attention recently. This is a ferroelectric perovskite with a wide area of applications [1]. Its interesting properties result from its different forms, depending on the temperature. Within the temperature range of 0–130 °C, the ferroelectric tetragonal phase of BaTiO_3_ is stable, and when the temperature is above 130 °C, it shows stability in a paraelectric cubic phase [2]. Particularly interesting is the use of barium titanates for the fabrication of capacitors or ultracapacitors. For example, Reynolds et al. reported on the application of sputtered modified BaTiO_3_ for the preparation of thin-film capacitors. The authors proved that such capacitors were characterized by dielectric loss and a significant leakage [3]. Next, Wodecka-Duś et al. presented ceramic materials based on lanthanum-doped barium titanate with good electrical permittivity that may be applied for the fabrication of ultracapacitors [4]. The high electrical permittivity of ferroelectric BaTiO_3_ was noticed also by Wu et al., who applied the microparticles of this compound for the preparation of filaments applied for fused deposition modeling, i.e., one of the 3D printing techniques. Applied filaments consisted of polymer matrix based on acrylonitrile butadiene styrene and barium titanate microparticles, which were introduced into such a matrix. As result, microwave devices characterized by a high dielectric permittivity have been printed [5].

Barium titanates have also found applications in the preparation of piezoelectric devices. For example, Wei et al. described investigations on the sintered piezoelectric ceramic based on barium titanate. This inorganic compound has been processed using extrusion free-forming from milled precursors. As a result, ceramic material with promising piezoelectric and dielectric properties has been obtained [6]. Barium titanate has also been used for the fabrication of silicone elastomers to form dielectric elastomers with good permittivity and mechanical properties [7]. Another application has been proposed by Uttan et al. They carried out investigations on a discotic liquid crystalline material prepared using ferroelectric nanoparticles of barium titanate. The introduced BaTiO_3_ nanoparticles affected the thermal stability of the material and caused the decrease of its permittivity [8]. Next, Giannakoudakis et al. proposed the deposition of the homogeneous coating formed from barium titanate nanospheres on the carbon textile fibers. They reported that such a formed composite may be useful as an efficient protection material against the toxic vapors [9]. Another use of barium titanate was described by Yang et al. They formed a core–shell filler based on BaTiO_3_ and nickel hydroxide and dispersed such a filler in the poly(vinylidene fluoride–hexafluoropropylene) matrix [10]. Additionally, barium compounds are used for optical measurements [11].

The ferroelectric properties of barium titanate have been also taken into account during the designing of the materials fabricated for the energy storage application [12,13,14] or the energy conversion [15]. On the other hand, barium titanate has also found wide application in medicine, e.g., in tissue engineering [16,17,18,19,20,21]. Different methodologies for the fabrication of barium titanate/hydroxyapatite scaffold, e.g., multistage spark plasma sintering [22], hydrothermal process [23], or pulsed laser deposition [24], have been investigated.

In the case of preparation methods of barium titanate, the mechanochemical synthesis of this compound is the research subject of many investigations. Such a methodology may reduce the size of the particles obtained and allows receiving nano-sized powders [25]. Such a synthesis was presented by Lazarevic et al. [26], Sydorchuk et al. [27], and Ohara et al. [28]. Many investigations have been also performed on the suspensions of barium titanate [29,30] and the stability of such suspensions [31,32].

BaTiO_3_ is most widely used in making multilayer ceramic capacitors (MLCC), thermistors, and piezoelectric sensors. The inkjet printing (IJP) process can be used to fabricate MLCC of complex configurations by integrating internal electrodes and dielectric layers in a single step using a multi printing-head system. Stabilized aqueous suspensions of BaTiO_3_-based powders are required to obtain dielectric inks adapted to IJP. There is no information in the scientific literature on stabilized solutions of barium titanate prepared by mechanochemical synthesis. One of the characteristic features of the ceramic material obtained in this way is its fragmentation and a large number of structural defects, which may result in different properties in water suspensions. One of the goals of the research is to explore the possibility of preparing stabilized suspensions of BaTiO_3_ powders made by unconventional synthesis method using high-energy milling devices.

In the research, barium titanate has been synthesized using BaO and TiO_2_ subjected to mechanochemical grinding. Subsequently, the obtained perovskite powder has been dispersed in superabsorbent polymer suspension (SAP) based on acrylic poly(ethylene glycol), and the process was supported by microwave radiation. Final suspensions differed in the concentration of SAP applied. Both prepared perovskite powder and obtained dispersions have been investigated. The process of perovskite synthesis was evaluated using XRD analysis, its structure was characterized by Fourier Transform Infrared (FT-IR) spectroscopy, the morphology was assessed by SEM technique, and the specific surface area was determined by Brunauer, Emmett and Teller (BET) physical adsorption isotherms. Next, investigations on the impact of SAP concentrations on the pH, sedimentation process, and the dynamic viscosity of the suspension formed were performed. Additionally, the pH and zeta potential measurements of aqueous suspension of barium titanate have been performed.

## 2. Materials and Methods

### 2.1. Materials

Barium oxide (powder, 97%), potassium hydroxide (powder), acrylic acid (99%, d = 1.051 g/mL, anhydrous, contains 200 ppm Mequinol (MEHQ) as inhibitor), ammonium persulfate (98%), poly(ethylene glycol), and *N*,*N*′-methylenebis(acrylamide) were bought from Sigma Aldrich (Saint Louis, MO, USA) TiO_2_ (powder, 98%) was bought from Evonik Degussa P25 GmbH (Essen, Germany).

### 2.2. Applied Methodology

**Figure 1:**
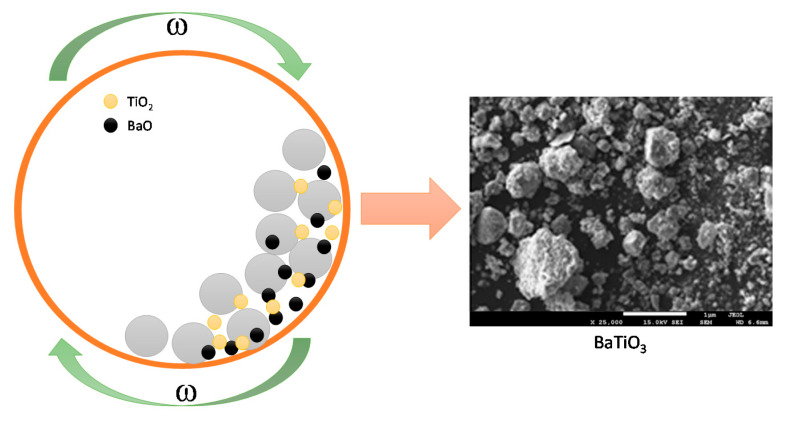
The scheme of the preparation of BaTiO_3_ using a ball mill.

In order to receive BaTiO_3_ with a homogeneous structure, the oxides used for its preparation were first heat treated to remove hydroscopic water and then mixed before grinding in a stoichiometric ratio using an agate mortar. Next, such prepared mixture was mechanochemically treated in a high-energy planetary ball mill Activator 2S (Activator Corporation, Novosibirsk, Russia). Milling was conducted at room temperature in air. A 250 mL reaction vessel was used, along with Cr-Ni steel ball bearings of 10 mm in diameter. The vessel was rotated at 1100 rpm for 1.5 h, with a ball-to-powder weight ratio (BPR) equal 40:1 [33,34,35]. The scheme of the preparation of barium titanate BaTiO_3_ has been presented in Figure 1 below.

**Figure 2:**
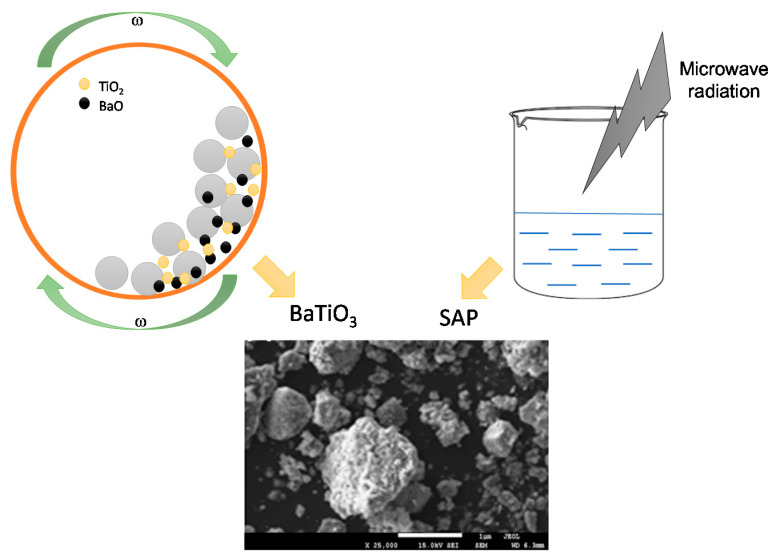
The scheme of the preparation of the dispersion of BaTiO_3_ stabilized by superabsorbent polymer suspension (SAP).

The acrylic poly(ethylene glycol) suspensions (SAP) were prepared using microwave irradiation. The appropriate amount of acrylic acid, potassium hydroxide, and poly(ethylene glycol) were mixed. Subsequently, the crosslinking agent (i.e., *N*,*N*′-methylenebisacrylamide) and initiator (i.e., ammonium persulfate) were introduced. Finally, the reaction mixture was treated with microwave irradiation (reactor produced by Milestone Srl., power 600 W, Sorisole, Italy) for 3 min. The temperature of the reaction mixture was maintained between 90 and 95 °C. The SAP suspension obtained was characterized by a solid, very viscous, and uniform jelly-like structure [33,34,35]. The scheme of the preparation of the dispersion by mixing adequate amounts of BaTiO_3_ and acrylic poly(ethylene glycol) suspension of SAP is shown below in Figure 2.

During the syntheses, the amount of barium titanate used was constant, while the amounts of SAP applied was changed. The compositions of the obtained dispersions are presented in Table 1.

Prepared perovskite powders as well as the obtained dispersion of barium titanate in SAP were subsequently characterized using various methods.

Firstly, the phase composition of barium titanates was identified by X-ray Diffractometry using a Philips X’Pert diffractometer (Eindhoven, The Netherlands) with Cu Kα radiation (λ = 0.15418 nm) and an Ni filter (Philips, Eindhoven, The Netherlands) equipped with graphite monochromator PW 1752/00 operating at 30 kV and 30 mA. The diffraction patterns were recorded in the 2θ range from 10° to 90° with a step size of 0.01°. The material was identified by comparing the obtained patterns with Join Committee for Powder Diffraction Standards (JCPDS) data.

The structure of the obtained powder was characterized by Fourier transform infrared spectroscopy. Such a study was carried out to identify the product of the performed process and determine the functional groups in its structure. Investigations were conducted by a Spectrum 65 (Perkin Elmer, Waltham, MA, USA) spectrometer that was equipped with an attenuated total reflection (ATR) attachment with a diamond/ZnSe crystal. The FT-IR spectra were recorded within the range of 3500–500 cm^−1^ (32 scans, 4.0 cm^−1^ resolution), and the analysis was carried out at room temperature.

Next, the surface area of perovskite powders received was determined by BET (Brunauer, Emmett and Teller) physical adsorption isotherms. The study was conducted using Accelerated Surface Area and Porosimetry of Micrometrics Analyzer (ASAP) 2020.

The surface morphology of BaTiO_3_ received was characterized by a JEOL JSM 7500F SEM (JEOL Ltd., Tokyo, Japan) microscope equipped with an energy-dispersive (EDS) detector (voltage 10 kV). Samples before analysis were coated with gold (this element was not included in the elemental analysis). EDS analysis was performed to determine the elemental composition of the powder received.

The stability of barium titanate particles in SAP suspension was determined by sedimentation investigations. For this purpose, each suspension was transferred into test tubes, and the initial suspension heights (h_o_) were determined. Next, after 1 and 24 h, the sediment heights (h) were measured again. The h/h_o_ ratios were presented as a function of the SAP concentrations.

The stability of suspensions received was determined by pH measurements conducted after 10 days from the synthesis as a function of SAP concentration (50%, 67%, 75%, 80%, and 83%, respectively) and the incubation time (24 h, 72 h, 120 h, and 168 h, respectively). Additionally, analysis of the pH and zeta potential of aqueous suspension of barium titanate (3% wt.) was performed.

Finally, the dynamic viscosities of the suspensions formed were also measured. The analyses were performed at ambient temperature by means of an Anton Paar CV-2 PP viscosimeter with mandrel R2.

## 3. Results and Discussion

### 3.1. Analysis of Barium Titanate Powder Using X-ray Diffraction (XRD) Technique

The synthesis of barium titanate proceeds according to the following reaction (Equation (1)):

BaO + TiO_2_ → BaTiO_3_ (ΔG = −158 kJ/mol). (1)

**Figure 3:**
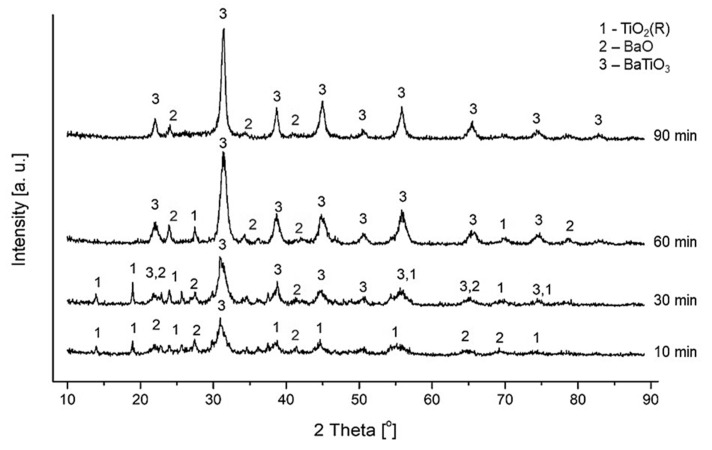
XRD patterns of mechanochemically synthesized barium titanate.

After 1.5 h of high-energy ball milling of TiO_2_ and BaO substrate powders, a product consisting of barium titanate was received. The structural changes of such treated powders were determined by XRD technique after 10 min, 30 min, 60 min, and 90 min, respectively. The obtained XRD patterns are presented in Figure 3.

As it may be noticed in Figure 3, 10 min of high-energy mechanochemical treatment of the mixture of BaO and TiO_2_ powders lead to the beginning of the formation of crystalline barium titanate. In the first minutes of milling, the disappearance of most diffraction reflections that correspond to the substrates phases is observed. Visible diffraction reflections are characterized by exceptionally low intensity. This is due to the high fragmentation of grains and their partial amorphization. After 30 min of high-energy ball milling, small diffraction reflection at 2Theta angles of 22.18, 31.6, 38.94, 45.26, and 50.95 are visible, indicating the formation of the crystalline BaTiO_3_ phase. With further milling, most of the diffraction reflections of the substrates disappear. After 90 min of high-energy processing of powders, mainly the barium titanate cubic phase (JCPDS 31-174) is observed. A small amount of a secondary BaO phase was observed in the ceramics.

### 3.2. Analysis of the Structure of Obtained Powder by Fourier Transform Infrared (FT-IR) Spectroscopy

**Figure 4:**
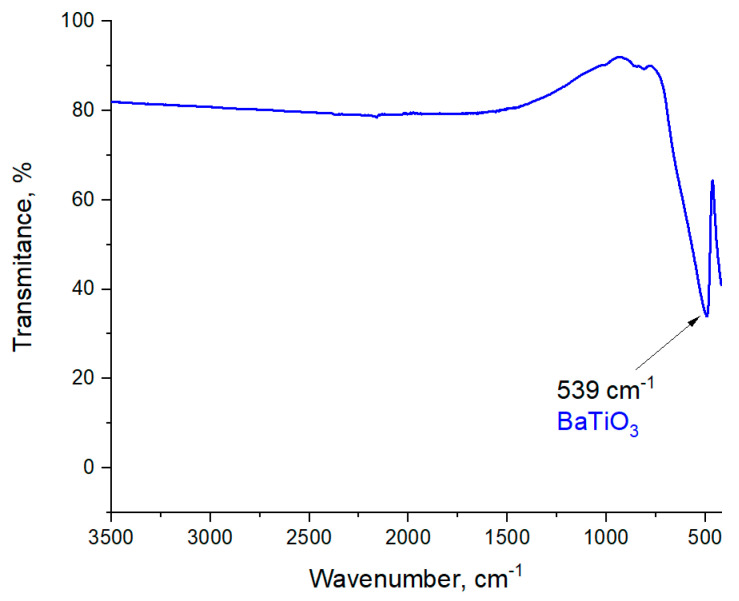
Fourier Transform Infrared (FT-IR) spectrum of barium titanate.

In Figure 4, the FT-IR spectrum of the obtained powder is shown.

The absorption peak visible at approximately 539 cm^−1^ corresponds to the stretching vibrations of Ti–O. Such a peak indicates the formation of barium titanate [36,37].

### 3.3. Determination of the Specific Surface Area of the BaTiO_3_ Powders

The specific surface area is defined as the size of the external surface area of the solid substance per mass of this substance that was presented using the equation below.

(2) $$\sigma_{m}\overset{\mathrm{def}}{=}\frac{S}{m}$$

where:

S—the external surface area of the solid substance.
m—the mass of the substance.

Such a defined specific surface area is expressed in m^2^/kg or m^2^/g.

In Table 2, the results of the BET analysis are presented.

The specific surface area of barium titanate was 25.275 m^2^/g (Table 2), which was determined by BET adsorption isotherm measurements. Such a value may be determined as low for such types of materials. Comparing the previous results of the research presented in Ref. [33], the value of the specific surface area determined for calcium titanate CaTiO_3_ obtained also by means of the treatment using a ball mill was 38.14 m^2^/g. Such a value also has been evaluated as a low one for this type of material.

Further investigations involved analyses using scanning electron microscope that were performed to obtain better images of the surface of the materials received.

### 3.4. Scanning Electron Microscope with Energy Dispersive X-ray (SEM-EDS) Spectroscopy

**Figure 5:**
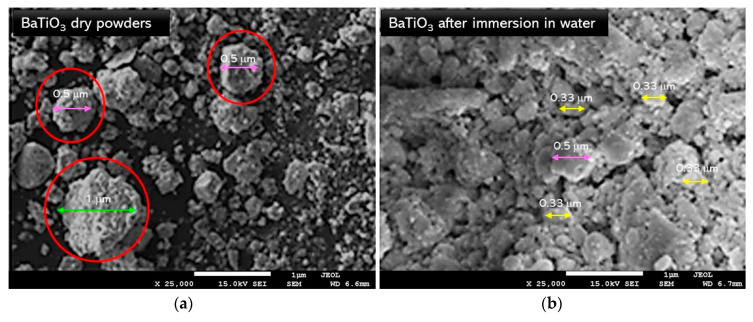
SEM microphotographs of mechanochemically obtained BaTiO_3_ powders in dry state (**a**) and after immersion in water (**b**).

**Figure 6:**
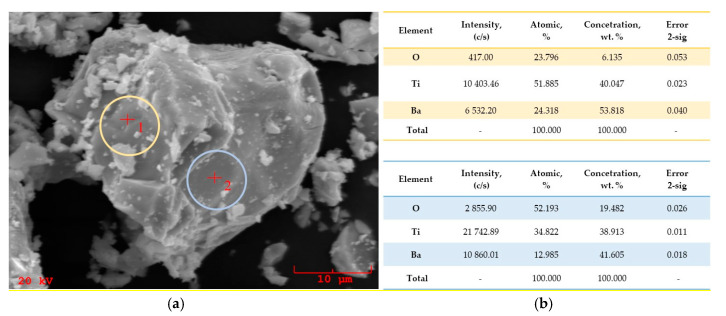

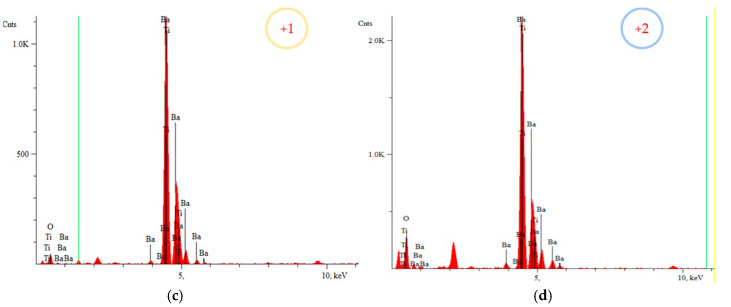
SEM-EDS points analysis: SEM image of the powder obtained (**a**), the summary of the content of the specific chemical elements (**b**) and the EDS spectrum in point 1. (**c**) and point 2. (**d**) marked on the SEM image.

**Figure 7:**
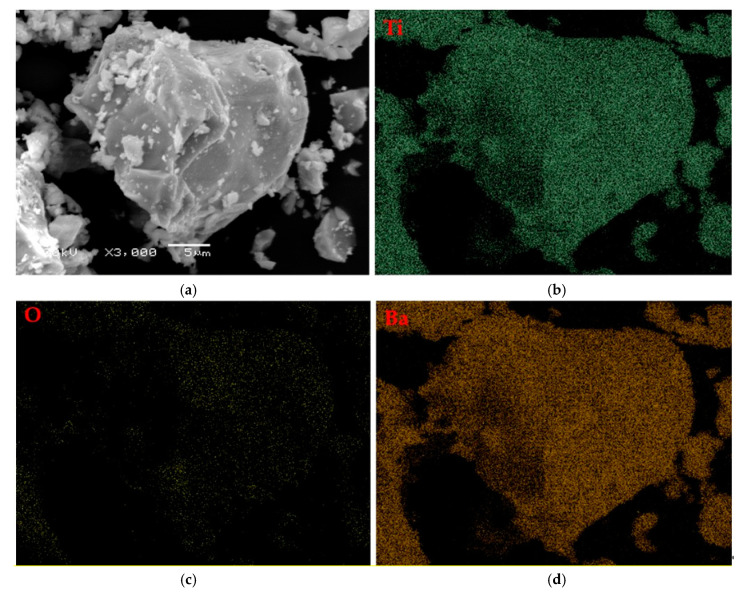
SEM−EDS elemental mapping for BaTiO_3_: SEM image (**a**) and the corresponding SEM mapping of titanium (**b**), oxygen (**c**) and barium (**d**).

**Figure 8:**
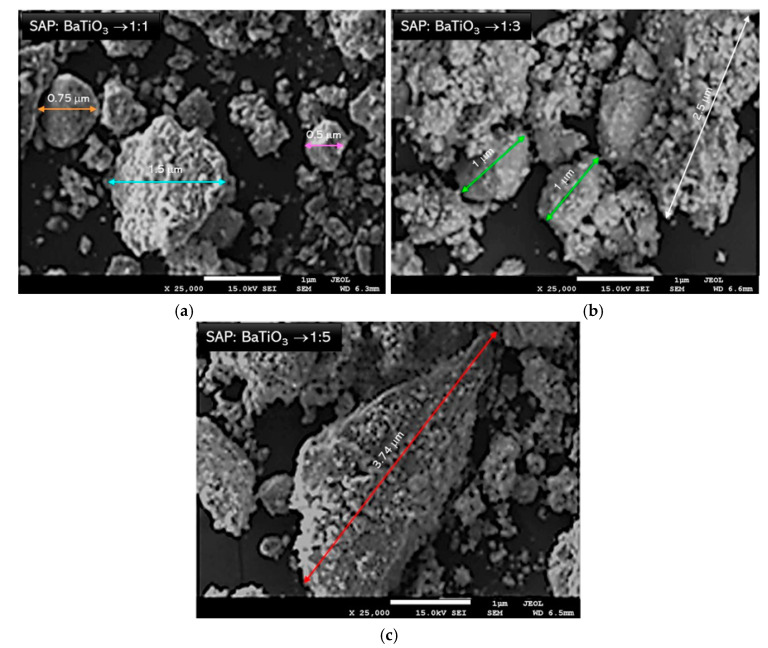
SEM images of SAP suspensions of barium titanates with different mass ratio of SAP and BaTiO_3_, i.e., 1:1 (**a**); 1:3 (**b**) and 1:5 (**c**).

The research was carried out to determine the morphology and the surface of attained powders. SEM images of BaTiO_3_ in a form of a dry powder and after the immersion of water are presented in Figure 5. In Figure 6 and Figure 7, SEM-EDS results, including the analysis of the elemental composition and the elemental mapping of tested powder in selected points, have been shown. In turn, in Figure 8, SEM images of barium titanates stabilized by SAP suspensions are shown. Additionally, the size of particular grains has been determined.

Analyzing Figure 5a, it may be observed that the obtained barium titanate powders were characterized by a grainy structure, wherein the grains were micro- and nanometric. The morphology of barium titanates grains after their immersion in water was also determined, which is presented in Figure 5b. Based on the images obtained, it may be concluded that after the immersion in water, the presence of the smallest particles that were visible previously, i.e., in the figure presenting BaTiO_3_ in a dry state—were not reported. This phenomenon is probably related to the process of aggregation of barium titanate particles in the water environment (this has also been confirmed during the sedimentation investigations presented in further on in this paper). Such an aggregation may affect the sedimentation rate.

Performing EDS analysis allows stating that the elemental composition of the tested barium titanate particles includes elements such as barium, oxygen, and titanium. Any other element has not been observed.

Detailed analysis of the SEM images presented in Figure 8 allowed concluding that SAP suspensions resemble a rather dry form of barium titanate compared with the aqueous suspension. This may be because SAP may act as a stabilizing agent, which prevents the aggregation of barium titanate particles. Furthermore, comparing all the images presented in Figure 8, it may be concluded that any significant impact of the increasing amount of SAP in the dispersion on the structures obtained was not observed. The received structures are comparable. Therefore, it may be stated that the SAP concentration is not a factor determining the size and the shape of the particles obtained.

### 3.5. Investigations on the Sedimentation Process of Barium Titanate Particles in Obtained Suspenions

**Figure 9:**
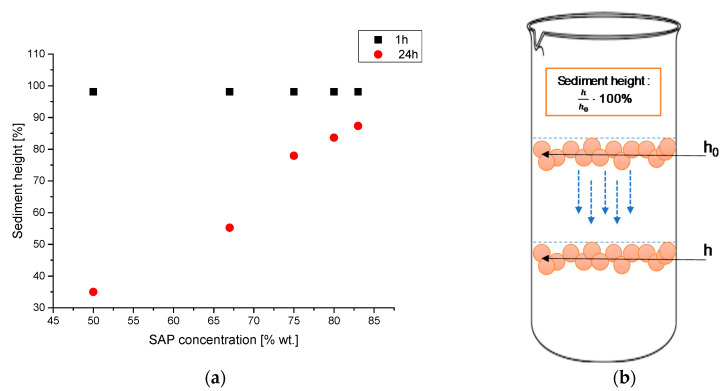
The sediment heights as a function of the SAP concentration in the tested suspensions (**a**) and the scheme of the tested sedimentation process (**b**).

Suspensions containing various SAP/BaTiO_3_ mass ratios were prepared as described in Section 2.2 of this paper. All the samples were tested to determine the stability of suspensions. For this purpose, the measurements of the sediment heights were conducted after a certain period of time, i.e., after 1 h and 24 h after the synthesis, respectively. In Figure 9, the changes in the sediment height as a function of SAP concentration in the tested suspensions are presented as well as the scheme of the sedimentation process being analyzed.

In Figure 9a, the correlation between the SAP concentrations in suspensions with BaTiO_3_ as a function of sediment height is presented. Measurements after 1 h of the incubation remained constant. The sedimentation height reached a value equal to 98.1%, independently of the SAP concentration in any particular dispersion. On the other hand, sediment heights determined after 24 h were significantly influenced by SAP concentrations.

The investigations showed that dispersions with a higher values of SAP mass fraction exhibited lower sedimentation rates. It was noticed that for samples containing 83 wt.% of SAP (BaTiO_3_:SAP = 1:5), relatively large sediment heights were observed, reaching values equal to 87.3%. Moreover, concentrations of SAP affected the density of the precipitates; i.e., the higher the SAP concentration, the lower the precipitate density. Particles included in the stable dispersions of BaTiO_3_ tended to sediment very slowly. According to the Stokes law of sedimentation, bigger particles settle faster than the smaller ones, which float freely in the suspension, leading to the formation of a non-transparent solution above the precipitate.

Furthermore, a higher concentration of polymer solution (SAP) resulted in an enhanced dispersion of barium titanate. Importantly, the presented results of the investigations on SAP dispersions of barium titanate remain in agreement with the results of previously presented analyses of SAP dispersions of calcium titanate [35]. This may lead to a more general conclusion that all perovskite titanates behave in a similar manner when dispersed in SAP.

### 3.6. pH Investigations

In the figures below, results of the pH measurements are presented. Analyses were conducted from the viewpoint of the changes of SAP concentrations (pH was measured 10 days after the synthesis) and the time (pH was measured directly after the synthesis).

**Figure 10:**
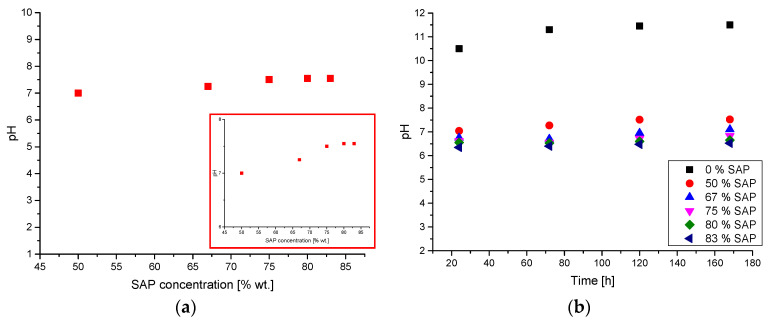
Changes in the pH values of BaTiO_3_ suspensions as a function of SAP concentrations (**a**) and incubation time (**b**).

The stability and lower sediment density of suspensions with higher SAP concentrations is correlated with pH values measured. As it may be noticed in Figure 10a, the pH value depends on the SAP concentration. Strict correlation between pH and SAP concentration shows that with increasing SAP concentrations, the pH value is also slightly increasing. Importantly, higher pH values favor the formation of porous sediment structures with large spaces among agglomerates. Variable SAP concentrations only slightly affect the pH values. What is more, the increase in the SAP concentration from 50% wt. to 83% wt. does not significantly change the total acidity of the suspension.

Based on the results presented in Figure 10b, analyses of the pH changes of the aqueous solution of BaTiO_3_ without the addition of SAP show that the pH is changing from 10.5 to 11.5. Comparison of the pH values of the SAP suspensions of BaTiO_3_ and the aqueous solution of BaTiO_3_ indicates the great differences between the pH values during the whole period of the research. The aqueous solution of barium titanate without the addition of SAP possesses a pH equal to 10.5, whereas the remaining mixtures upon the addition of SAP reach a pH value near 6.0–7.0, which indicates the strong influence of the introduced SAP on pH value. Moreover, the increase in SAP concentration does not significantly change the pH value of the suspension. Small differences in the pH values may be noticed among 50% wt. of SAP and the other concentrations.

#### Measurement of pH and Zeta Potential of Aqueous Suspension of Barium Titanate

**Figure 11:**
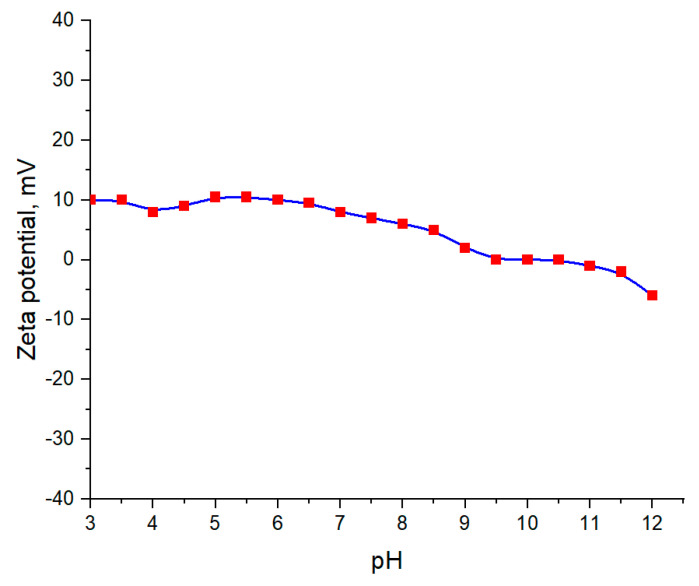
Measurements of pH and zeta potential of aqueous suspension of BaTiO_3_ (without SAP).

Figure 11 shows the pH and zeta potential measurements of BaTiO_3_ suspension in distilled water.

As can be seen in Figure 11, the pH of an aqueous suspension of barium titanate is alkaline. This is a result of the hydrolysis of BaTiO_3_ that proceeds according to the following Equation:

(3) $${\mathrm{BaTiO}}_{3}+H_{2}O↔{\mathrm{Ba}}^{2+}+{\mathrm{TiO}}_{2}+2{\mathrm{OH}}^{-}.$$

The release of OH^−^ ions results in the increase of the pH of the suspension and also in the decrease of the zeta potential [38]. For pH equal to approximately 10.0, the zeta potential decreases, achieving the isoelectric point (IP). Such a big value in which the IP is achieved is caused by the presence of OH^−^ ions and the simultaneous appearance of barium ions in the suspension. Similar conclusions were drawn by Chiang et al. [39].

### 3.7. Investigations on the Dynamic Viscosity

**Figure 12:**
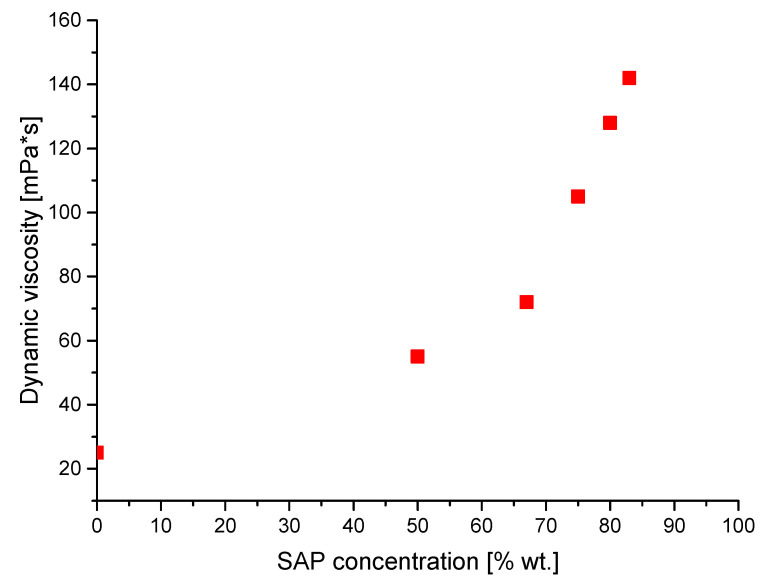
Dynamic viscosity as a function of SAP concentration in BaTiO_3_ suspension.

In Figure 12, the impact of the SAP introduction into the barium titanate suspension on the viscosity of obtained suspension is presented.

The viscosity value is crucial from the viewpoint of determining the potential applications of the tested suspension. Moreover, suspensions with too low or too high viscosities cause difficulties in handling and during their applications or storage. The change of dynamic viscosity as a function of SAP concentration is shown in Figure 12. The minimum value of viscosity is assigned to the aqueous solution of BaTiO_3_ without the SAP addition. It may be concluded that the increase in SAP concentration leads to the increase of dynamic viscosity. A low viscosity of the suspension may indicate that the distances between molecules in such a medium are large, and therefore, particles can move freely. On the contrary, an increase of the viscosity results in a reduction of the distances between particles, and thus their mobility is diminished and may lead to friction among particles. Molecules of stabilizing agents such as SAP may adsorb to the surface of particles and thus prevent their agglomeration according to the rules of steric and electrostatic effects. As a result, BaTiO_3_ particles dispersed in SAP are well monodispersed and have sediments with lower speeds, as was observed in sedimentation experiments whose results were presented in Figure 7. This effect can be explained by the Stokes–Einstein law, according to which the higher viscosity of the dispersing phase leads to the lower rates of particles sedimentation. Therefore, variable changes of SAP concentrations, and hence changes of dispersion viscosities, can be used as a regulating agent for particles settling.

## 4. Conclusions

The mechanochemical process proceeding with the use of a ball mill is a suitable technique applied for successful synthesis of perovskites such as barium titanate.

The full conversion of mechanically grinded BaO and TiO_2_ powders toward barium titanate occurs after 90 min of milling, which was proven by the crystallographic analysis of the obtained powder. Moreover, the band at 539 cm^−1^ is characteristic for the stretching vibrations of Ti–O and was observed on an obtained FT-IR spectrum that also confirmed the formation of barium titanate. Furthermore, EDS analysis confirmed that in tested samples there was only barium, oxygen, and titanate.

SAP suspensions of BaTiO_3_ more closely resemble the dry powder of barium titanate than aqueous suspension.

Analysis of the suspension properties exhibited no significant changes of pH with the time after the synthesis. On the contrary, the dynamic viscosity of the suspension was increasing with the addition of SAP.

SAP can prevent the aggregation of the particles in colloidal suspensions by electrostatic repulsions between the hydrolyzed acidic groups, or OH groups form a polymer surfactant conjugated to the surface of particles that also limits the formation of their aggregates. Such a conclusion suggests that SAP in examined suspensions constitutes an excellent stabilizing agent and prevents precipitation or sedimentation of the mineral phase.

These conclusions stay in line with conclusions drawn based on the previous studies of the systems based on calcium titanates [33]. This finding may suggest that groups of perovskite materials such as titanates behave in a similar manner when dispersed in acrylates.

## Figures and Tables

**Table 1:** Composition of the BaTiO_3_ suspension stabilized by SAP.

BaTiO_3_ [g]	Distilled Water [mL]	SAP [g]	SAP [wt.%]
1.0	100	0	0
		1.00	50
		2.00	67
		3.00	75
		4.00	80
		5.00	83

**Table 2:** The specific surface area of BaTiO_3_ after 90 min of the synthesis proceeded in a ball mill.

Compound	Specific Surface Area, m^2^/g
BaTiO_3_	25.275
